# Calcium application coordinates seedling heat tolerance and yield-quality formation in ginger under summer high-temperature conditions

**DOI:** 10.3389/fpls.2026.1921048

**Published:** 2026-10-06

**Authors:** Xingcui Wang, Xiumei Ni, Xiaoming Liu, Zhiyu Li, Hongshen Guo, Qiqin Xue, Chunlei Li

**Affiliations:** 1 Shandong Facility Horticulture Bioengineering Research Center, Weifang University of Science and Technology, Shouguang, China; 2 Anqiu Ministry of Agriculture and Rural Affairs, Anqiu, China

**Keywords:** antioxidant enzymes, calcium nutrition, natural high-temperature stress, PCA, rhizome quality, *Zingiber officinale*

## Abstract

Summer heat stress constrains ginger production. Although calcium (Ca) enhances stress tolerance in various crops, its effects on heat tolerance, yield, and quality in ginger remain unclear. A pot experiment was conducted using *Zingiber officinale* cv. ‘Laiwu Big Ginger’ to evaluate Ca effects on plant growth and quality under natural summer high-temperature conditions. Six Ca rates (0, 15.2, 30.5, 45.7, 60.9, and 76.2 mmol Ca^2+^ plant^-1^) were established using calcium nitrate tetrahydrate [Ca(NO_3_)_2_·4H_2_O]. Seedling-stage physiological and biochemical parameters were analyzed with linear mixed-effects models; harvest-stage growth, biomass, and quality parameters were analyzed with ANOVA or non-parametric tests. PCA, correlation analysis, and membership function analysis were used for multivariate evaluation. At Day 16, the 60.9 mmol Ca^2+^ plant^-1^ treatment significantly improved photosynthetic capacity (chlorophyll +23.3%, Pn +13.0%), antioxidant enzyme activities (SOD, POD, CAT), and proline content, while reducing oxidative damage (MDA, REC) and ROS (O_2_^·-^, H_2_O_2_). These adjustments promoted biomass and rhizome quality, with the highest rhizome fresh weight (727.7 vs. 447.3 g plant^-1^), dry matter (+3.6%), soluble sugars (+28.1%), soluble protein (+36.5%), free amino acids (+19.1%), and vitamin C (+17.5%). Multivariate analyses confirmed positive correlations among photosynthetic traits, antioxidant activity, biomass, and quality parameters, with the 60.9 mmolCa^2+^ plant^-1^ treatment ranking highest in comprehensive evaluation by membership function analysis. Calcium application at this optimal rate, as determined under our experimental conditions, was associated with enhanced heat tolerance and subsequent growth and quality formation. These findings provide practical insights for sustaining ginger productivity under heat stress within the scope of our experimental conditions.

## 1 Introduction

Temperature is a key regulatory factor governing plant growth and morphogenesis by modulating core physiological metabolism processes such as water transport, enzymatic reactions, and photosynthesis (Karim et al., 2000). It also regulates the biosynthesis and accumulation of internal metabolites, thereby influencing the quality of edible organs and agricultural products (Chaudhary et al., 2022). When ambient temperature exceeds the optimal growth threshold of plants, normal physiological metabolism is disrupted, leading to physiological disorders that ultimately restraining plant growth and development (Feng et al., 2023). With ongoing climate change, rising temperatures pose a significant threat to the yield and quality of horticultural crops worldwide. Substantial evidence indicates that heat stress triggers excessive reactive oxygen species (ROS) production, which disrupts chloroplast biogenesis, accelerates chlorophyll degradation, and ultimately reduces photosynthetic efficiency and biomass accumulation (Wang et al., 2018; Fortunato et al., 2023; Mathur et al., 2014; Gururani et al., 2015). Concurrently, high temperatures modify plant morphology, physiology, and enzymatic activities, accelerate phenological progression, shorten the vegetative-to-reproductive transition, and negatively impact crop productivity, nutritional status, and quality (Allakhverdiev et al., 2008; Hu et al., 2020; Chaudhary et al., 2022).

Ginger (*Zingiber officinale* Roscoe) is a perennial herbaceous species of the family Zingiberaceae (Varakumar et al., 2017). Its rhizomes possess both medicinal and culinary values, making it an economically important crop widely cultivated worldwide. The optimal daily mean temperature for ginger growth ranges from 20 to 28 °C. When ambient temperatures exceed 35 °C, physiological functions, vegetative growth, and rhizome swelling are severely inhibited (Liu et al., 2023). Although Z. *officinale* is a thermophilic and shade-loving species, its seedlings are particularly vulnerable to heat injury during summer, when daily maximum temperatures consistently surpass the optimal range and often peak above 39 °C, especially when combined with intense solar radiation (Gong et al., 2023). Such conditions result in stunted plants, reduced yield, and deteriorated quality. Notably, while ginger prefers warm conditions, it is also highly sensitive to temperatures above its optimum and susceptible to chilling injury. Previous studies have indicated that thermophilic herbaceous species are generally more sensitive to high-temperature stress and prone to physiological heat damage (Chaudhary et al., 2022). The entire growth cycle of ginger spans the summer season, during which the seedling stage—the most stress-sensitive phase—coincides with the period of extreme high temperatures, inflicting significant physiological damage.

As an essential macronutrient, calcium contributes to multiple physiological processes that underpin plant growth and stress tolerance, including preserving membrane integrity, stabilizing cell wall structures, regulating ion transport and selectivity, and modulating cell wall enzyme activities (Marschner, 1995). Ca^2+^ not only sustains normal cellular activities, but also functions as an intracellular second messenger (White and Broadley, 2003)abolic homeostasis in response to environmental changes and stresses (Zhang et al., 2022; Ravi et al., 2023; Feng et al., 2023). Previous studies have established that exogenous calcium plays positive roles in crop growth, development, and quality formation under heat stress. Appropriate calcium application has been shown to effectively mitigate heat stress-induced damage in several horticultural crops, including common bean (Naeem et al., 2020), rose (Wang et al., 2022), and *Dendrobium* (Fan et al., 2023). Such mitigation is evidenced by reduced ROS production, enhanced activities of antioxidant enzymes, increased leaf pigment content, and improved photosynthetic efficiency (Feng et al., 2023). In ginger specifically, exogenous calcium has been shown to alleviate heat stress through a combination of physiological strategies, including osmotic adjustment, upregulation of antioxidant enzyme activities, accumulation of osmolytes (sodium, potassium, and proline), and stimulation of root development (Liu et al., 2023; Gong et al., 2023).

In recent years, heatwaves have become increasingly frequent and intense. The warming effect inside solar greenhouses further exacerbates heat stress on horticultural crops under protected cultivation. Studying how calcium alleviates this stress during extreme heat years could provide theoretical basis for adaptive cultivation strategies under future climate warming. Although exogenous calcium has been documented to mitigate heat stress in various horticultural crops, including tomato (Tuna et al., 2007; Siddiqui et al., 2017; Ayub et al., 2022), common bean (Naeem et al., 2020) and orchid (Fan et al., 2023), its role in ginger remains unexplored. Moreover, most existing studies are confined to short-term physiological responses at the seedling stage, leaving a critical gap in understanding whether calcium can confer sustained benefits across the entire growth cycle—from vegetative development to rhizome maturation—and whether it can simultaneously enhance final yield and quality. Given that the entire growing season of ginger coincides with recurring summer heatwaves, and its seedlings are particularly vulnerable to supra-optimal temperatures, addressing these questions is both scientifically imperative and practically relevant.

To address this gap, we used ginger (*Z. officinale* cv. ‘Laiwu Big Ginger’) as the experimental material and investigated, for the first time, the mechanisms by which exogenous calcium enhances thermotolerance in seedlings under high-temperature conditions, while extending our evaluation to harvest-stage growth and quality traits. Through comprehensive assessments of heat injury alleviation, antioxidant defense, and osmotic regulation during the seedling stage, combined with measurements of plant biomass, rhizome yield, and nutritional quality at harvest, we aimed to determine the optimal calcium application rate and to assess whether calcium-induced seedling thermotolerance can translate into synergistic improvements in both production and quality. Our findings are expected to provide a theoretical basis and actionable technical parameters for the safe summer cultivation of ginger under intensifying global warming.

## 2 Materials and methods

### 2.1 Plant materials and growth condition

Plant materials were grown in pots under greenhouse conditions at Weifang University of Science and Technology, Shandong, China, in 2023. The greenhouse was naturally ventilated with side and roof vents kept fully open during the summer hot period to promote air circulation, and fitted with a 50% shading net that was continuously applied from the seedling stage to the vigorous vegetative growth stage and removed during the rhizome bulking stage. *Zingiber officinale* Roscoe (cv. ‘Laiwu Big Ginger’) was used as the experimental material, which has a growth cycle from April to October. Seed rhizomes were sprouted and planted when the bud length reached 1-1.5 cm. Each pot (40 cm × 40 cm × 30 cm) was filled with 15 kg of air-dried and sieved garden soil and one seed rhizome. The soil was a sandy loam (determined by the hydrometer method). The garden soil was air-dried, sieved through a 5-mm mesh, and thoroughly homogenized before potting. The fundamental physical and chemical properties of soil were as follows: pH 7.41, organic matter 12.9 g·kg^-1^, alkaline-hydrolyzable nitrogen 62.5 mg·kg^-1^, available phosphorus 7.75 mg·kg^-1^, available potassium 43.6 mg·kg^-1^, and exchangeable calcium 2.7 g·kg^-1^ (determined by ammonium acetate exchange-AAS method). Pots were uniformly spaced to avoid mutual shading.

Air temperature inside the greenhouse was continuously monitored using automatic temperature and humidity data logger (TPJ-20, Top Cloud-Agri, Hangzhou, China) with sensors placed 15 cm above the pot canopy, recording at 30-min intervals. Concurrent outdoor temperature data were obtained from the Shouguang Meteorological Bureau. In July 2023, the average maximum temperature in Shouguang City was 34 °C, while the daily maximum temperature inside the greenhouse was on average 3-5 °C higher than outdoors, creating a more severe stress environment. During the sampling period (July 5-21), the daily maximum temperature inside the greenhouse ranged from 33.2 to 41.5 °C (based on TPJ-20 continuous monitoring data), with the highest value of 41.5 °C recorded on July 6; over the same period, the outdoor daily maximum temperature ranged from 31 to 38 °C. The heat stress intensity (HSI) was calculated using the following formula $\text{HSI}=\sum_{\text{i}=1}^{\text{n}}\left(\text{Ti-}35{}^{\circ}\text{C}\right)$, where Ti is the daily maximum temperature, and only days with Ti > 35 °C were included. The HSI for July 2023 was 20 °C·d, which was significantly higher than the mean value of 13.0 ± 11.3 °C·day for the same period from 2020 to 2024 (temperature data derived from daily observations of the Shouguang Meteorological Bureau). Consequently, the ginger plants were exposed to prolonged supra-optimal temperatures during the seedling stage, which confirms that a natural heat stress event occurred throughout the experimental period. Although relative humidity was not continuously recorded, periodic spot checks using a handheld thermo-hygrometer indicated that it generally remained within 60%–85% during the high-temperature period. Basal fertilizers were applied at a rate of 6 g N, 3 g P_2_O_5_, and 7 g K_2_O plant^-1^, and standard agronomic practices were uniformly implemented across all growing seasons.

It should be emphasized that this experiment was conducted under naturally fluctuating high-temperature conditions inside the greenhouse, rather than under artificially controlled constant temperatures. During the experimental period, daily maximum temperatures fluctuated, with intermittent intervals below 35 °C, which realistically represents the field temperature regime that ginger seedlings experience during summer heatwaves.

### 2.2 Experimental design

In this experiment, six fertilization treatments were established: CK (0 mmol Ca^2+^ plant^-1^), T1 (15.2), T2 (30.5), T3 (45.7), T4 (60.9), and T5 (76.2). Calcium nitrate tetrahydrate [Ca(NO_3_)_2_·4H_2_O] was used as the calcium source. Each experiment treatment contained four replicates, which were arranged following a completely randomized design. In total, 20 pots were assigned to each treatment, with 5 pots allocated per replicate. All pots were randomly positioned within the greenhouse, and their positions were re-randomized weekly to minimize positional effects. Calcium nitrate was applied in three splits: 20% as basal fertilizer (incorporated into the soil before planting), 40% as seedling fertilizer (applied on the afternoon of July 4), and 40% during rhizome bulking (applied on September 1). At each application, the designated amount of Ca(NO_3_)_2_·4H_2_O was dissolved in 200 ml of water and evenly poured around the base of each plant, avoiding foliar contact. To maintain uniform total nitrogen availability across treatments, urea was used to regulate for nitrogen variability caused by differing Ca(NO_3_)_2_ application.

Irrigation was managed as follows: routine irrigation was applied throughout the entire growth period to maintain moist soil conditions. During the high-temperature monitoring period in July, moisture control was achieved using the gravimetric method. Prior to this period, the field water capacity of the soil was determined using the cutting-ring method, and the target pot weight (including pot, soil, and plant fresh weight) corresponding to 75%-80% of field water capacity was calculated for each pot. During the monitoring period, all pots were weighed every two days, and water was replenished based on the difference between the actual weight and the target weight. Water was applied slowly and uniformly using a sprinkling can to avoid runoff along the pot wall or leakage from the bottom. After the monitoring period, routine irrigation management was resumed.

### 2.3 Index determination

The three-fork stage of ginger signals the start of vigorous vegetative growth and is highly sensitive to heat stress. This stage occurs in July, coinciding with the summer high-temperature period. All treatments were fertilized (application as a seedling topdressing) on the afternoon of July 4. July 5, when the daily maximum temperature inside the greenhouse first stably exceeded 35 °C, was set as the baseline day (day 0) for heat stress monitoring, with subsequent sampling at 4-day intervals, resulting in five time points: 0, 4, 8, 12, and 16 d. It should be noted that during the measurement period, the daily maximum greenhouse temperature fluctuated naturally, with intermittent cooling episodes below 35 °C. Therefore, the heat conditions in this experiment represent natural fluctuating high temperatures, rather than artificially controlled constant heat stress.

For all physiological, biochemical, morphological, and quality parameters, each biological replicate was measured in duplicate (two technical replicates), and the average value was used for statistical analysis. For biomass parameters (shoot fresh weight, root fresh weight, and rhizome fresh weight), each biological replicate was measured once due to the destructive nature of sampling.

#### 2.3.1 Photosynthetic physiological indices

Determination of chlorophyll (Chl) content and net photosynthetic rate (Pn): Chlorophyll was extracted from fully expanded functional leaves using 80% acetone, Chlorophyll a (Chl a) and b (Chl b) content was quantified by spectrophotometry according to the method of Lichtenthaler (1987).

Consistent with the protocol of Long et al. (1996), we measured net photosynthetic rate (Pn) with a CIRAS-2 portable photosynthesis system between 9:00 and 11:00 a.m.

#### 2.3.2 Hydrogen peroxide and superoxide anion radical content

H_2_O_2_ content was determined using the titanium sulfate colorimetric method following the protocol of Brennan and Frenkel (1977).

O_2_^•−^ production rate was measured using the nitroblue tetrazolium (NBT) reduction method as described by Ramel et al. (2009).

#### 2.3.3 Malondialdehyde content and relative electrolyte conductivity index

The MDA content was detected through the thiobarbituric acid (TBA) reaction method proposed by Hodges et al. (1999).

The REC was tested in accordance with the method described by Wang et al. (2022).

#### 2.3.4 Enzyme activities and proline content

To evaluate oxidative stress in leaves, we measured the activities of superoxide dismutase (SOD), peroxidase (POD), and catalase (CAT) in adult leaves. Mature leaves were collected from four selected ginger plantlets per treatment, immediately frozen in liquid nitrogen, and stored at -80 °C until analysis. Two independent technical replicates were performed for each biological replicate.

For enzyme extraction, frozen leaf tissue (0.5 g) was homogenized in 5 mL of ice-cold 50 mM phosphate buffer (pH 7.8) containing 1% (w/v) polyvinylpyrrolidone (PVP). The homogenate was centrifuged at 12,000 × g for 20 min at 4 °C, and the supernatant was used as the crude enzyme extract for activity assays (Madamanchi et al., 1994).

SOD activity was determined by measuring its ability to inhibit the photochemical reduction of nitroblue tetrazolium (NBT) at 560 nm (Kamran et al., 2021). POD and CAT activities were assayed spectrophotometrically by monitoring the oxidation of guaiacol at 470 nm and the decomposition of H_2_O_2_ at 240 nm, respectively, following the method of Kamran et al. (2021).

Proline (Pro) content was quantified with the acid-ninhydrin method in accordance with the protocol reported by Bates et al. (1973).

#### 2.3.5 Morphological and biomass index

On the harvest day, four representative plants with uniform growth were selected from each treatment. We measured plant height from the soil surface up the growing point of the primary stem, and recorded stem diameter at 1 cm above ground level. The plants were gently uprooted, immediately rinsed with tap water, and surface-dried using filter paper. Each ginger plant was cut and separated into shoots, roots, and rhizomes. The number of green leaves per plant was counted, and fresh weights of the separated parts were recorded individually using an electronic balance. The fresh weight of rhizomes per plant was calculated as the average value of total biomass of each treatment.

#### 2.3.6 Dry matter and quality indices

Leaf and rhizome samples were first heated at 105 °C in an oven for 30 min to deactivate enzymes, and then dried at 80 °C to constant weight (approximately 48 h). The leaves were used for pigment content determination, while the dry weights of rhizomes were measured and recorded using an electronic balance to calculate the dry matter content. For quality assays, 0.5 g of rhizome tissue of the first lateral shoot was sampled from four randomly selected plants per treatment. The content of soluble sugar (SS) was detected via the anthrone-sulfuric acid colorimetric method (Zhang et al., 2006). The content of soluble protein (SP) was tested with the Coomassie Brilliant Blue G-250 dye-binding assay (Bradford, 1976). Free amino acid (FAA) content was analyzed via ninhydrin colorimetry (Troll and Cannan, 1953). Ascorbic acid (AsA) content was determined using the 2,6-dichlorophenolindophenol sodium titration (method, following the protocol described by Sairam et al. (2002). Crude fiber (CF) content was estimated according to AOAC Official Method 978.10 (Horwitz, 2000).

### 2.4 Statistical analysis

Data was sorted in Microsoft Excel 2019, and all statistical analysis was performed using SPSS 26.0. For the physiological and biochemical indicators measured repeatedly during the seedling stage (photosynthetic characteristics, ROS, membrane damage, antioxidant enzymes, and osmolytes), linear mixed-effects models were applied to account for correlations among repeated measurements. The models included time as a fixed factor, calcium rate and its quadratic term as covariates, individual plant as random intercept, and variance components (VC) as the covariance structure. *Post-hoc* multiple comparisons were conducted using estimated marginal means with Bonferroni correction.

For growth, biomass, and quality parameters measured at harvest (single time point), data were first tested for normality using the Shapiro–Wilk test and for homogeneity of variances using Levene’s test. When both assumptions were satisfied, one-way ANOVA followed by Duncan’s multiple range test was performed to assess differences among treatments (P ≤ 0.05). When data met normality but not homogeneity of variances, Welch’s ANOVA was used for group comparisons, followed by Games-Howell *post-hoc* tests. When the normality assumption was violated, the non-parametric Kruskal–Wallis test with Bonferroni correction was employed for pairwise comparisons.

To eliminate temporal fluctuations and comprehensively reflect the dynamic trends of each physiological and biochemical index during the seedling stage, during which natural high-temperature conditions occurred, the trapezoidal rule was applied to calculate the area under the curve (AUC) for the five time-point measurements. The AUC values were used to represent the cumulative physiological response of each treatment at this stage. Pearson correlation coefficients were calculated to assess the synergistic relationships between the AUC values of physiological indices and plant growth parameters as well as quality indicators. The AUC was calculated as follows $\text{AUC}=\sum_{\text{i}}^{3}\frac{\left({\text{Y}}_{\text{i}}+{\text{Y}}_{\text{i}+1}\right)}{2}\times 4$, where Y_i_ is the measured value at the i-th time point t_i_ (i.e., t_i = 0, 4, 8, 12, 16 days for i = 0, 1, 2, 3, 4), with a constant interval of Δt = 4 days between consecutive measurements. The AUC values were used to represent the cumulative physiological response of each treatment at this stage. Before performing principal component analysis (PCA), the Kaiser–Meyer–Olkin (KMO) test and Bartlett’s test of sphericity were used to verify sampling adequacy and the correlation structure of the data. PCA was then performed to visualize variation patterns among treatments, and the results were presented as a clustered heatmap. Given the limited sample size, these analyses were interpreted with caution and are considered exploratory rather than confirmatory. PC1 scores derived from PCA were analyzed using one-way ANOVA followed by Tukey’s HSD *post-hoc* test for multiple comparisons among treatments. Finally, to comprehensively evaluate the overall effects of different fertilizer treatments on plant growth and quality, a membership function analysis was conducted. Only the growth parameters and quality indicators were selected for the calculation of the comprehensive evaluation value (D value), and the six Ca(NO_3_)_2_ gradients were ranked and evaluated based on their D values.

## 3 Results and analysis

### 3.1 Effects of exogenous calcium on photosynthetic characteristics of ginger leaves under natural high-temperature conditions

To investigate the effects of exogenous calcium on the photosynthetic system of ginger under naturally elevated temperatures, a linear mixed-effects models were applied to analyze total chlorophyll, chlorophyll a, chlorophyll b, and net photosynthetic rate (Pn), respectively.

Time had a highly significant main effect on total chlorophyll (F_(4, 84)_ = 670.953, P < 0.001), chlorophyll a (F_(4, 84.223)_ = 44.586, P < 0.001), chlorophyll b (F_(4, 84.204)_ = 2128.49, P < 0.001), and Pn (F_(4, 84.010)_ = 670.953, P < 0.001), with all parameters declining progressively as the duration of natural heat exposure extended (**Figures 1a–d**). The linear term of calcium rate significantly affected total chlorophyll (P < 0.001), chlorophyll b (P < 0.001), and Pn (P < 0.001), indicating that calcium application generally enhanced photosynthetic pigment contents and Pn.

**Figure 1:**
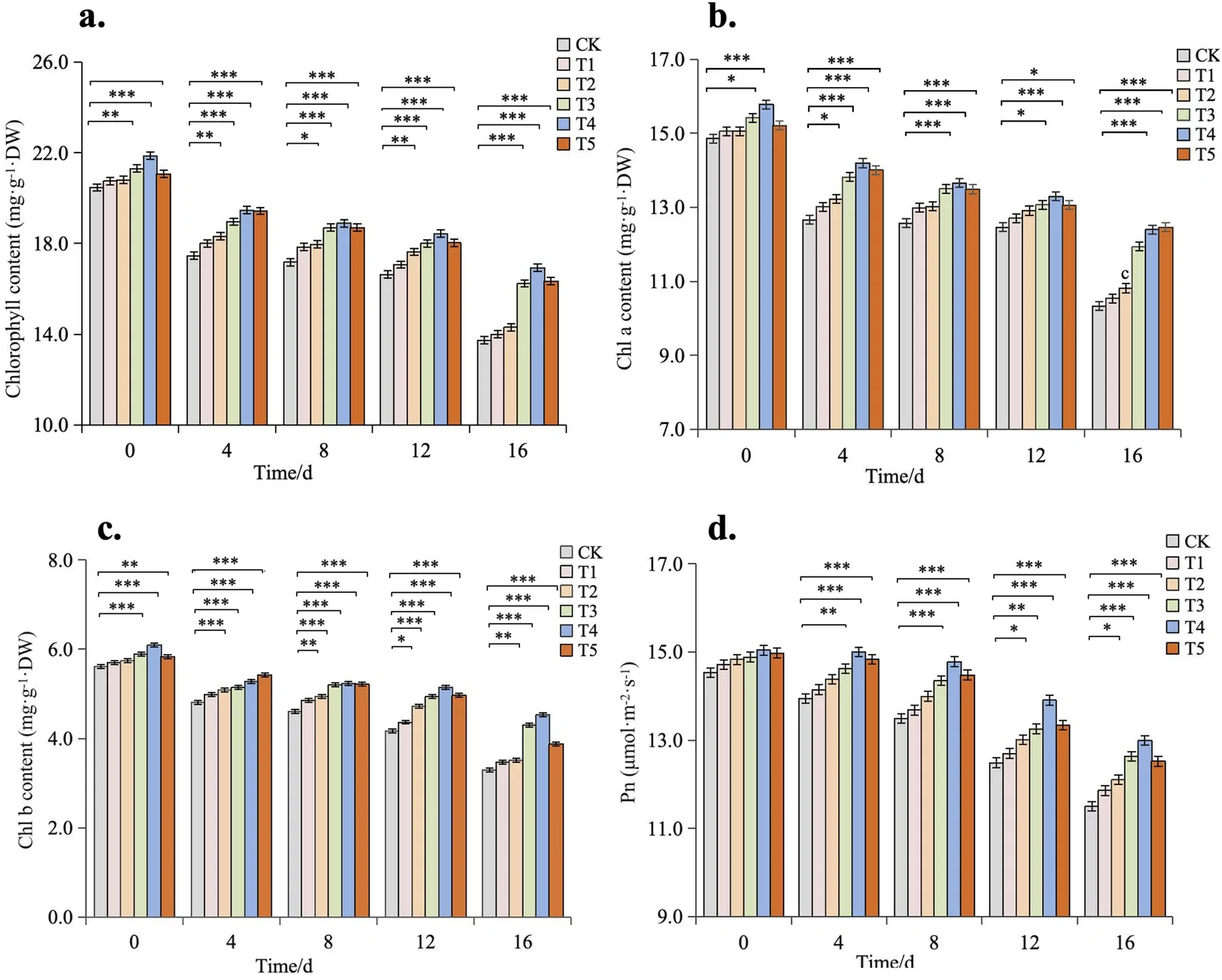
Effects of exogenous calcium on photosynthetic characteristics of ginger leaves under natural high-temperature conditions. **(a)** Total chlorophyll content; **(b)** chlorophyll a content; **(c)** chlorophyll b content; **(d)** net photosynthetic rate (Pn). Values are means ± SE (n = 4). *, **, and *** indicate significant differences from the CK treatment at P< 0.05, P< 0.01, and P< 0.001 respectively based on linear mixed-effects model with Bonferroni correction for repeated measurements.

Notably, the inverted U-shaped response to calcium was strongest for chlorophyll b (quadratic term estimate = −0.00261, P < 0.001), followed by total chlorophyll (estimate = −0.00283, P < 0.001), whereas chlorophyll a exhibited a significant inverted U-shape mainly at the mid-stage (12 d) of natural heat exposure (estimate = −0.006493, P = 0.039). The quadratic term for Pn was also significant (estimate = −0.00283, P = 0.004), and the time × quadratic calcium interaction was highly significant (F_(4, 83.992)_ = 4.717, P = 0.002). The fitted peaks for both photosynthetic pigments and Pn converged around 60.9 mmol Ca^2+^ plant^-1^(**Figures 1a–d**), jointly confirming the existence of an optimal calcium threshold for alleviating heat-induced damage.

Of particular interest, the protective effect on photosynthetic pigments was relatively sustained over time (remaining elevated from 8 to 12 d), whereas Pn declined earlier and more drastically (already substantially reduced by 12 d), revealing an apparent asynchrony in their temporal responses (**Figures 1a–d**). This suggests that calcium differentially regulates the structural and functional components of the photosynthetic system.

### 3.2 Effects of exogenous calcium on reactive oxygen species accumulation in ginger leaves under high-temperature conditions

To determine the extent of oxidative burst and the scavenging effect of calcium under high-temperature conditions, the same model was applied to analyze superoxide anion (O_2_^·^^-^) release rate and hydrogen peroxide (H_2_O_2_) content.

Time had a highly significant main effect on both O_2_^·^^-^ release rate (F(4, 84.077) = 1669.105, P < 0.001) and H_2_O_2_ content (F(4, 83.943) = 1421.147, P < 0.001), with both parameters accumulating progressively over the course of natural heat exposure (**Figures 2a, b**). The linear term of calcium rate significantly reduced both O_2_^·^^-^ (F(1, 21.009) = 22.015, P < 0.001) and H_2_O_2_ (F(1, 21.013) = 4.497, P = 0.046), indicating a general alleviating effect of calcium on ROS levels. Neither quadratic term was significant (P > 0.05), suggesting a linear declining response. The time × linear calcium interaction was significant for both O_2_^·^^-^ (F(4, 84.028) = 15.843, P < 0.001) and H_2_O_2_ (F(4, 83.890) = 3.385, P = 0.013), indicating that the scavenging efficiency of calcium varied over time (**Figures 2a, b**). Notably, calcium exerted a stronger scavenging effect on O_2_^·^^-^ (F = 22.015) than on H_2_O_2_ (F = 4.497), suggesting that calcium acts more directly and efficiently on the upstream ROS.

**Figure 2:**
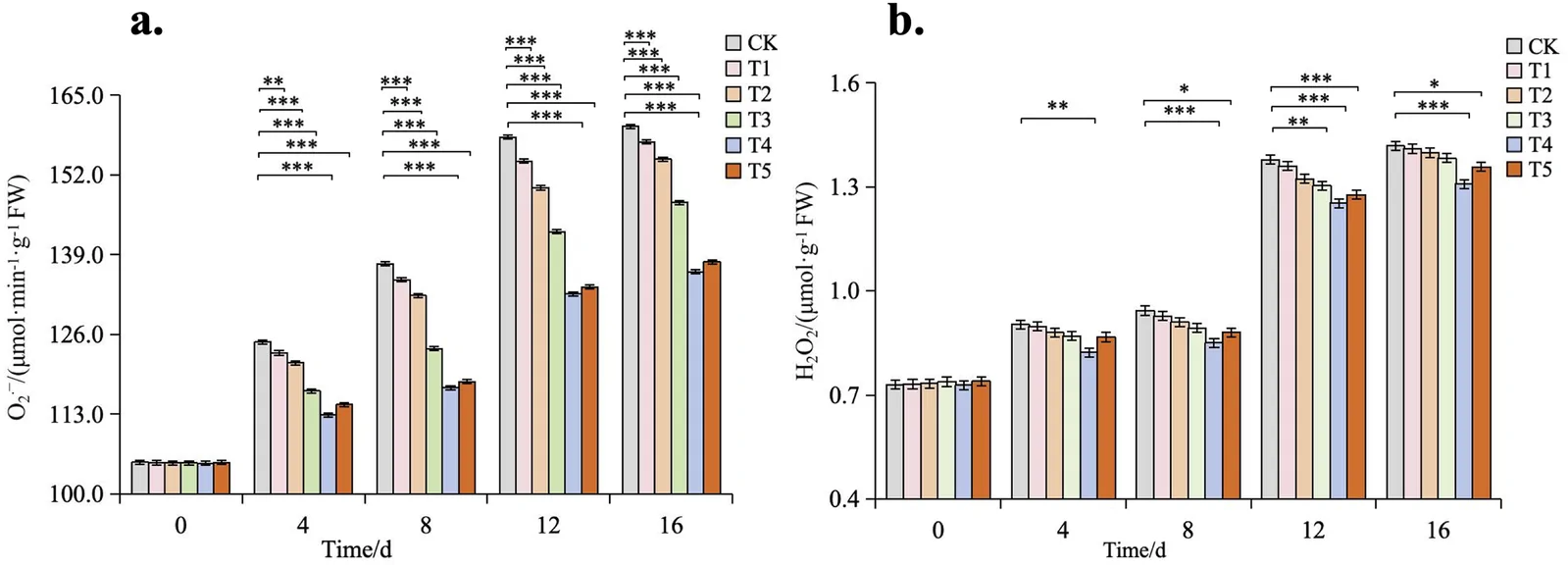
Effects of exogenous calcium on reactive oxygen species accumulation in ginger leaves under natural high-temperature conditions. **(a)** Superoxide anion (O_2_^·^^-^) release rate; **(b)** hydrogen peroxide (H_2_O_2_) content. Values are means ± SE (n = 4). *, **, and *** indicate significant differences from the CK treatment at P< 0.05, P< 0.01, and P< 0.001 respectively based on linear mixed-effects model with Bonferroni correction for repeated measurements.

### 3.3 Effects of exogenous calcium on membrane lipid peroxidation and membrane permeability in ginger leaves under natural high-temperature conditions

To evaluate the extent of membrane damage and the mitigating effect of calcium under natural high-temperature conditions, the same model was applied to analyze malondialdehyde (MDA) content and relative electrolyte conductivity (REC).

Time had a highly significant main effect on both MDA (F_(4, 84.043)_ = 1061.138, P < 0.001) and REC (F_(4, 84.037)_ = 1316.083, P < 0.001), with both parameters increasing progressively with extended natural heat exposure (**Figures 3a, b**). The linear term of calcium rate significantly reduced both MDA (F_(1, 21.013)_ = 41.187, P < 0.001) and REC (F_(1, 21.011)_ = 8.863, P = 0.007), indicating that calcium application effectively alleviated membrane damage.

**Figure 3:**
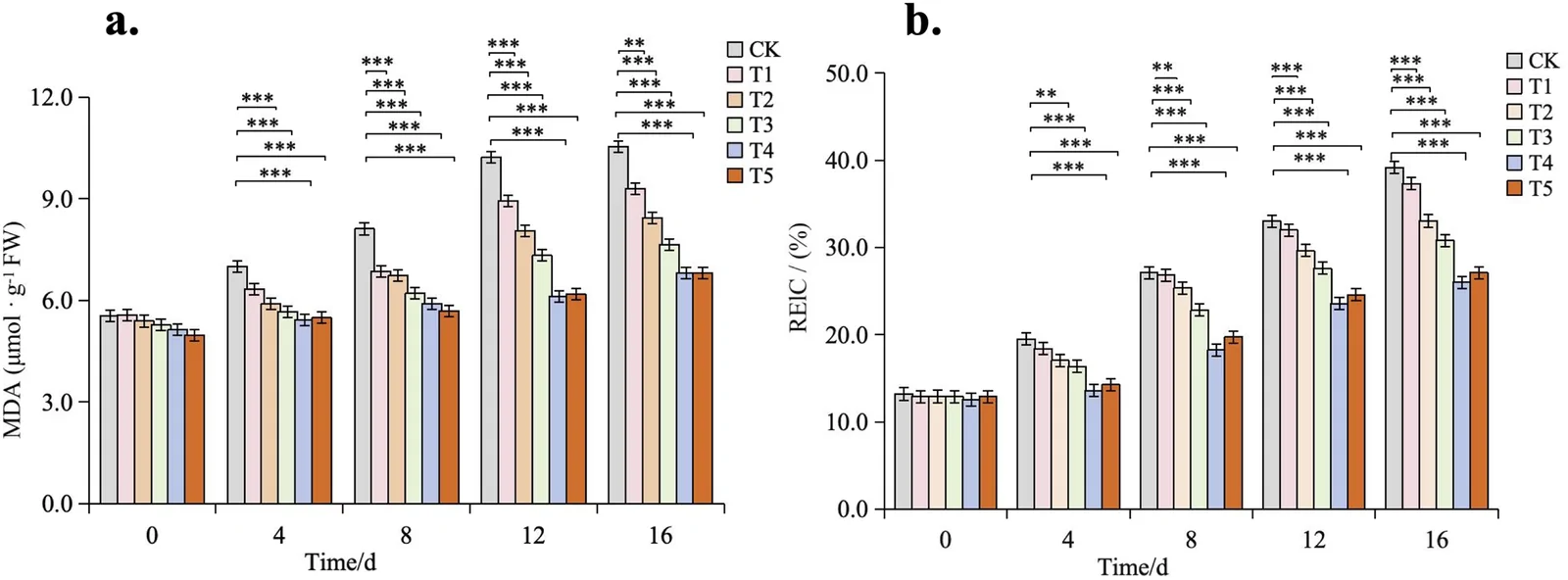
Effects of exogenous calcium on membrane lipid peroxidation and membrane permeability in ginger leaves under natural high-temperature conditions. **(a)** Malondialdehyde (MDA) content; **(b)** relative electrolyte conductivity (REC). Values are means ± SE (n = 4). *, **, and *** indicate significant differences from the CK treatment at P< 0.05, P< 0.01, and P< 0.001 respectively based on linear mixed-effects model with Bonferroni correction for repeated measurements.

The quadratic term of calcium rate was significant for MDA (F_(1, 21.014)_ = 7.800, P = 0.011), and the time × quadratic calcium interaction was highly significant (F_(4, 84.024)_ = 17.415, P< 0.001). For REC, the quadratic term alone was not significant (P = 0.611), but the time × quadratic calcium interaction was highly significant (F_(4, 84.020)_ = 3.928, P = 0.006), with parameter estimates showing negative and significant quadratic coefficients at 0, 4, 8, and 12 days, indicating a U-shaped response of REC to calcium rate at each time point (**Figure 3b**). Both parameters pointed to an optimal calcium rate of approximately 60.9 mmol plant^-1^, and this threshold effect was more pronounced during the early to mid-stage of natural heat exposure (0–8 d), gradually diminishing at later stages (12–16 d).

### 3.4 Effects of exogenous calcium on antioxidant enzyme activities and osmotic regulation in ginger leaves under natural high-temperature conditions

To evaluate the effects of exogenous calcium on the antioxidant defense system and osmotic adjustment capacity of ginger leaves under naturally elevated temperatures, the same mixed-effects model was applied to analyze the activities of SOD, POD, CAT, and Pro content.

Time had a highly significant main effect on SOD (F_(4, 84.032)_ = 201.738, P < 0.001), POD (F_(4, 84.035)_ = 29.211, P < 0.001), CAT (F_(4, 84.012)_ = 165.748, P < 0.001), and proline (F_(4, 84.084)_ = 9.017, P < 0.001), with temporal trends shown in **Figures 4a–d**. The linear term of calcium rate significantly enhanced SOD (F_(1, 21.008)_ = 13.944, P < 0.001), POD (F_(1, 20.988)_ = 18.046, P < 0.001), CAT (F_(1, 20.985)_ = 40.186, P < 0.001), and proline (F_(1, 20.993)_ = 7.413, P = 0.013), indicating that calcium application generally elevated enzyme activities and proline accumulation.

**Figure 4:**
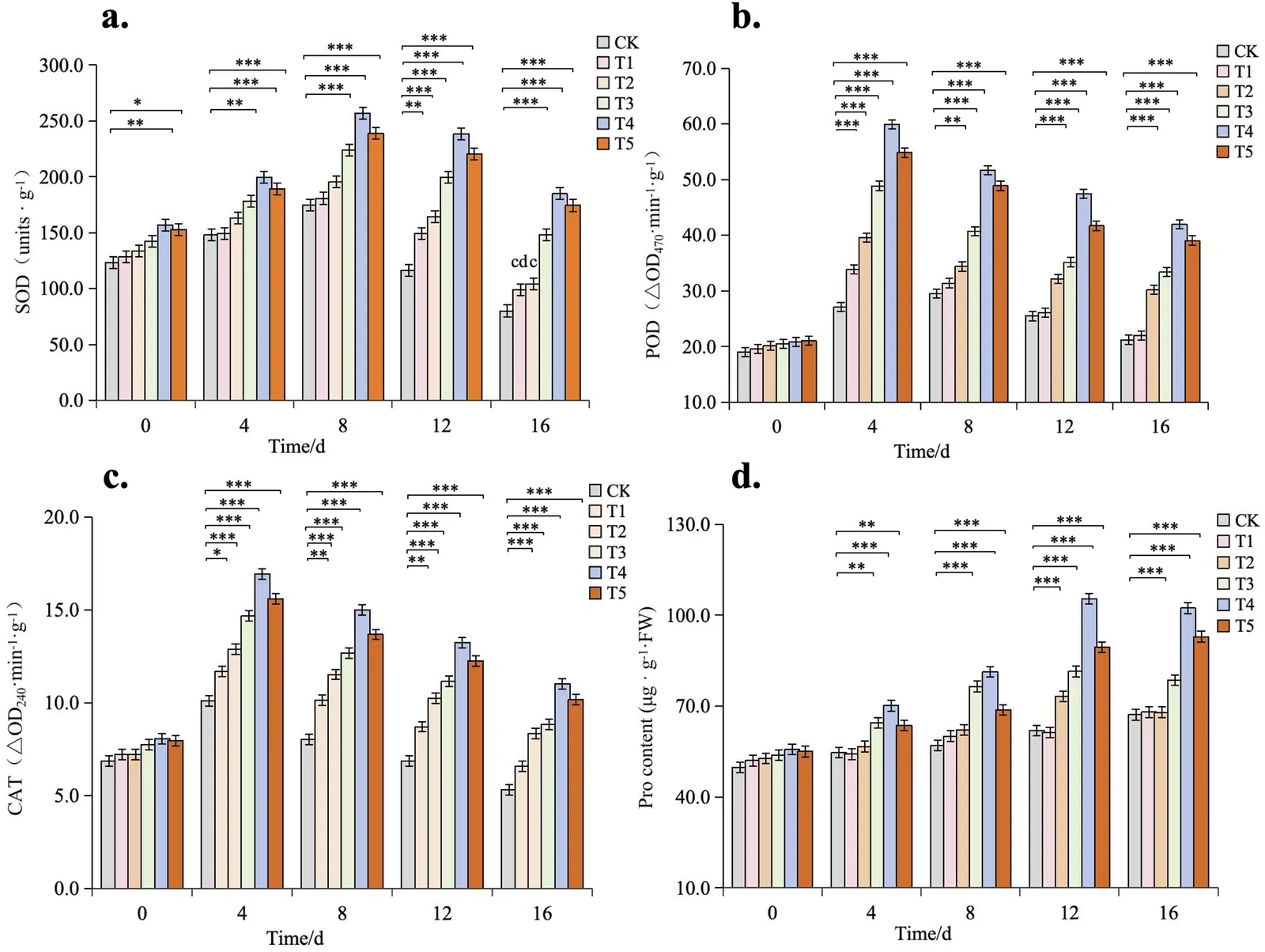
Effects of exogenous calcium on antioxidant enzyme activities and osmotic regulation in ginger leaves under natural high-temperature conditions. **(a)** Superoxide dismutase (SOD) activity; **(b)** peroxidase (POD) activity; **(c)** Catalase (CAT) activity; **(d)** Proline content. Values are means ± SE (n = 4). *, **, and *** indicate significant differences from the CK treatment at P< 0.05, P< 0.01, and P< 0.001 respectively based on linear mixed-effects model with Bonferroni correction for repeated measurements.

In terms of response patterns, the quadratic term of calcium rate was significant for CAT (F_(1, 20.985)_ = 8.649, P = 0.008; estimate = −0.000690), but not for SOD (P = 0.378) or POD (P = 0.396). However, the time × quadratic calcium interaction was highly significant for SOD (F_(4, 84.016)_ = 6.216, P < 0.001), POD (F(4, 84.004) = 5.624, P < 0.001), CAT (F(4, 83.995) = 12.304, P < 0.001), and proline (F_(4, 84.025)_ = 4.238, P = 0.004). Parameter estimates revealed that the quadratic coefficient for SOD was negative and highly significant at 12 d (estimate = −0.010756, P < 0.001); for POD, negative and significant at 4, 8, and 12 d (P = 0.049); for CAT, negative and significant at 8 d (P = 0.025); and for proline, negative and significant at 4, 8, and 12 d (P = 0.028, < 0.001, and 0.010, respectively). All four parameters exhibited an inverted U-shaped trend during the early to mid-stage of natural heat exposure (4–12 d), with the optimal calcium rate converging around 60.9 mmol plant^-1^ (**Figures 4a–d**).

Synthesizing all measured indicators, the alleviating effect of exogenous calcium on ginger under natural high-temperature conditions exhibited a highly consistent dose-dependent pattern: photosynthetic rate and chlorophyll content followed an inverted U-shape (initially increasing then decreasing), MDA and REC followed a U-shape (initially decreasing then increasing), and SOD, POD, CAT, and proline followed an inverted U-shape (initially increasing then decreasing). The optimal calcium rate for all four categories converged around 60.9 mmol plant^-1^. Moreover, the threshold effect for all indicators was most pronounced during the early to mid-stage of natural heat exposure (4–12 d) and gradually diminished or disappeared by 16 d, indicating a clear temporal window for calcium regulation. Collectively, these results systematically demonstrate, from multiple perspectives—including photosynthetic function, ROS metabolism, membrane stability, and antioxidant defense systems—the dose- and time-dependent characteristics of exogenous calcium in alleviating physiological damage in ginger under naturally elevated temperatures.

### 3.5 Effects of different Ca(NO_3_)_2_ rates on morphological and biomass parameters of ginger

Based on the data analysis strategy described in Section 2.4, one-way ANOVA was performed on each growth parameter at harvest, with results presented in **Figures 5a–f**.

**Figure 5:**
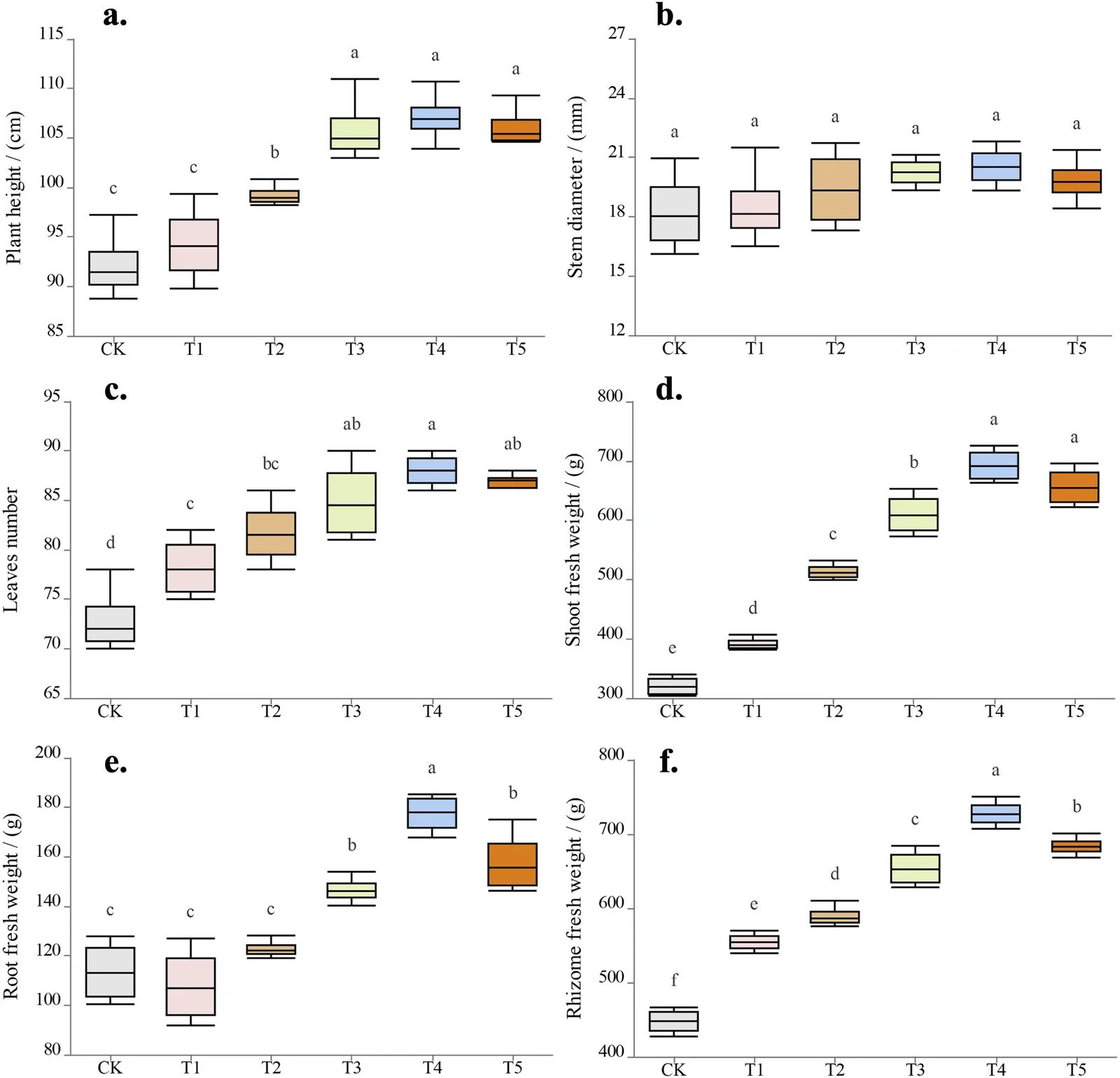
Effects of different Ca(NO_3_)_2_ rates on morphological traits and biomass accumulation of ginger. **(a)** Plant height; **(b)** Stem diameter; **(c)** Leaf number; **(d)** Shoot fresh weight; **(e)** Root fresh weight; **(f)** Rhizome fresh weight. Boxes represent the interquartile range (IQR) with the median line indicated inside; whiskers extend to the minimum and maximum values (n = 4). Different lowercase letters above boxes indicate significant differences among treatments at P< 0.05.

As shown in **Figure 5**, with increasing Ca(NO_3_)_2_ rates, all morphological and biomass parameters exhibited a clear trend of initial increase followed by a decline, with most parameters reaching their maximum values at T4 (60.9 mmol Ca^2+^ plant).

Regarding morphological traits, Ca(NO_3_)_2_ concentration had a highly significant effect on plant height (F = 17.996, df = 5, 18, P < 0.001). Duncan’s multiple range test revealed that plant height in T3 (105.95 cm), T4 (107.10 cm), and T5 (106.18 cm) was significantly greater than in CK (92.22 cm) and T1 (94.31 cm) (P < 0.05), with no significant differences among these three treatments; T2 (99.23 cm) was significantly higher than CK and T1 (P < 0.05), but significantly lower than T3, T4, and T5 (P < 0.05). Plant height was greatest in T4, representing a 16.1% increase over the control (**Figure 5a**). Unlike plant height, stem diameter did not differ significantly among treatments (F = 1.163, P = 0.365), with all treatments falling into the same homogeneous subset (**Figure 5b**), indicating that Ca(NO_3_)_2_ application had no notable promoting effect on stem diameter. Leaf number showed a similar promoting trend to plant height, with highly significant differences among treatments (F = 12.791, P < 0.001) and a highly significant linear increasing trend (F = 56.985, P < 0.001). Leaf number was highest in T4 (88.00 leaves), significantly greater than CK (73.00), T1 (78.25), and T2 (81.75) (P < 0.05), but not significantly different from T5 (86.50) (**Figure 5c**).

In terms of biomass accumulation, shoot fresh weight, root fresh weight, and rhizome fresh weight all followed patterns consistent with those observed for morphological traits. Specifically, shoot fresh weight differed highly significantly among treatments (Welch’s F = 133.314, P < 0.001), with T4 achieving the highest value (692.69 g), significantly greater than CK (320.35 g), T1 (391.74 g), T2 (513.38 g), and T3 (610.42 g) (P < 0.05), but not significantly different from T5 (656.54 g), representing a 116.2% increase over the control (**Figure 5d**). Root fresh weight also differed highly significantly among treatments (Welch’s F = 29.126, P < 0.001), with T4 reaching the highest value (177.25 g), significantly greater than CK (113.58 g), T1 (108.11 g), T2 (122.86 g), and T3 (146.62 g) (P < 0.05), but not significantly different from T5 (158.14 g), representing a 56.0% increase over the control (**Figure 5e**). As the core parameter for yield formation, rhizome fresh weight differed highly significantly among treatments (F = 125.313, P < 0.001), with a highly significant linear increasing trend (F = 545.347, P < 0.001). Rhizome fresh weight was highest in T4 (727.69 g), significantly greater than CK (447.32 g), T1 (554.35 g), T2 (589.57 g), and T3 (654.23 g) (P < 0.05), but not significantly different from T5 (683.65 g), representing a 62.7% increase over the control (**Figure 5f**). These results indicate that calcium nitrate significantly promoted biomass accumulation in both aboveground and belowground parts of ginger, with the relative increase in shoot fresh weight (116.2%) substantially exceeding that of total belowground fresh weight (roots + rhizomes, 61.3%).

### 3.6 Effects of different Ca(NO_3_)_2_ rates on rhizome dry matter and nutritional quality of ginger

Based on the data analysis strategy described in Section 2.4, one-way ANOVA was performed on each quality parameter at harvest, with results presented in **Figures 6a–f**.

**Figure 6:**
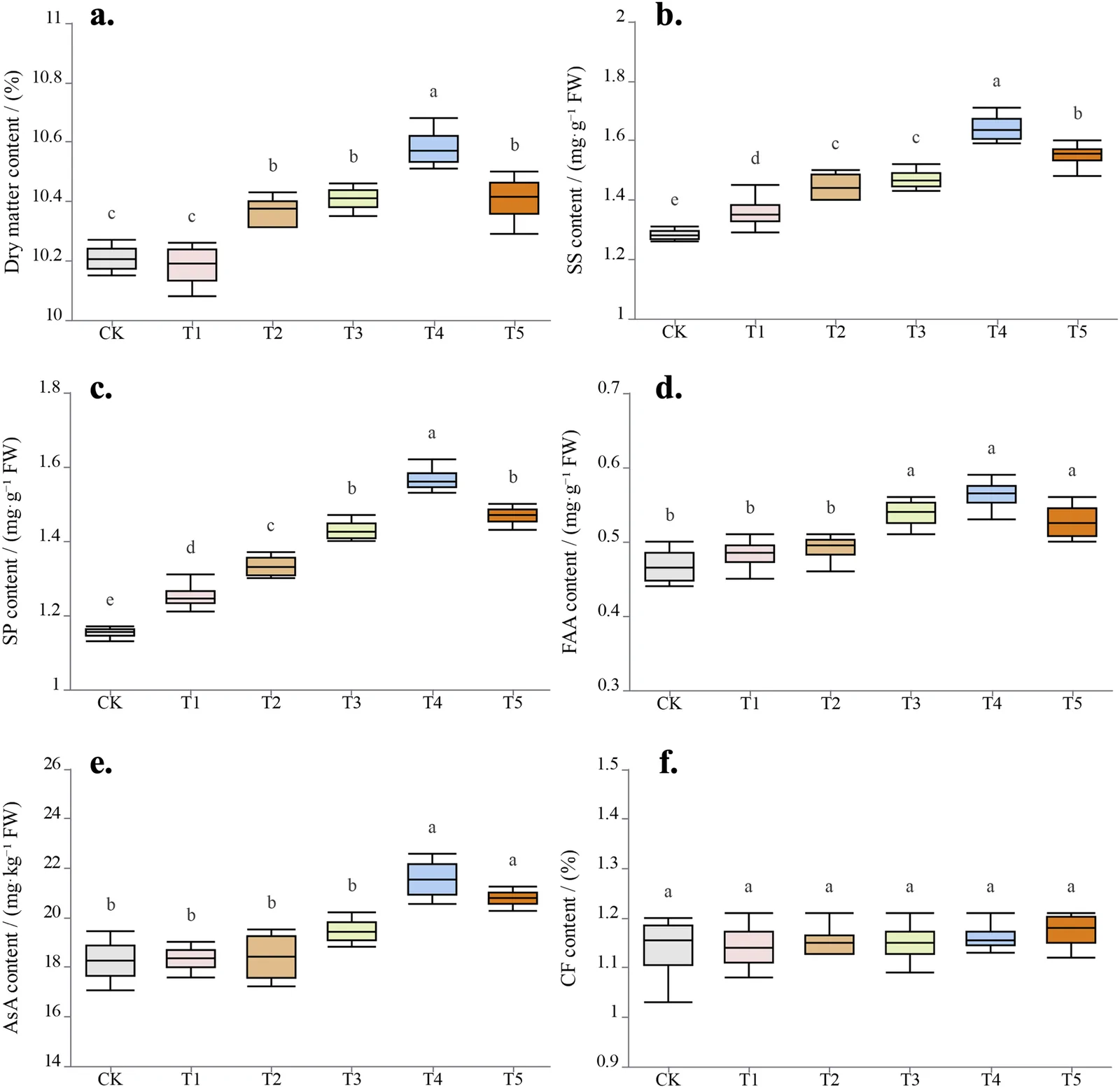
Effects of different Ca(NO_3_)_2_ rates on rhizome dry matter and nutritional quality of ginger. **(a)** Dry matter content; **(b)** Soluble sugar content; **(c)** Soluble protein content; **(d)** Free amino acid content; **(e)** Vitamin C content; **(f)** Crude fiber content. Boxes represent the interquartile range (IQR) with the median line indicated inside; whiskers extend to the minimum and maximum values (n = 4). Different lowercase letters above boxes indicate significant differences among treatments at P< 0.05.

As shown in **Figure 6**, with increasing Ca(NO_3_)_2_ rates, all quality parameters exhibited a clear trend of initial increase followed by a decline, with most parameters reaching their maximum values at T4 (60.9 mmol Ca^2+^ plant).

In terms of basic quality traits, dry matter content differed highly significantly among treatments (F = 13.523, P < 0.001), with a highly significant linear increasing trend (F = 45.178, P < 0.001). Dry matter content was highest in T4 (10.58%), significantly greater than CK (10.21%), T1 (10.18%), T2 (10.34%), and T3 (10.41%) (P < 0.05), but not significantly different from T5 (10.41%) (**Figure 6a**), indicating that the promoting effect of calcium fertilization resulted from effective dry matter accumulation rather than increased water uptake.

Regarding nutritional quality, soluble protein was the most responsive to calcium application (F = 84.289), with T4 showing a 36.5% increase over the control (P < 0.001) (**Figure 6c**). Soluble sugar content reached 1.64% in T4, significantly higher than all other treatments (P < 0.05), representing a 28.1% increase over the control (**Figure 6b**). Free amino acid content peaked in T4 (0.56%), significantly higher than CK (0.47%, P = 0.017) and T2 (0.49%, P = 0.034) (**Figure 6d**). Vitamin C content reached 2.15 mg g^-1^ in T4, significantly greater than CK (1.83 mg g^-1^), T1 (1.83 mg g^-1^), and T2 (1.84 mg g^-1^) (P < 0.05), representing a 17.5% increase over the control (Welch’s F = 11.691, P = 0.001) (**Figure 6e**).

Unlike the trends observed for the above quality parameters, crude fiber content did not differ significantly among treatments (F = 0.266, P = 0.926), with no significant differences between any treatment groups (P > 0.05) (**Figure 6f**), indicating that Ca(NO_3_)_2_ application did not cause rhizome lignification or textural deterioration.

### 3.7 Principal component analysis

In the preceding sections (3.1–3.6), the responses of individual indicators to calcium treatments were analyzed separately using mixed-effects models and one-way ANOVA. However, a single indicator is insufficient for a comprehensive evaluation of the overall effect of calcium application. To further elucidate the coordinated variation patterns among indicators and derive a quantitative metric for the integrated effect, principal component analysis (PCA) was performed in this section.

In the preliminary analysis, stem diameter showed no significant difference among treatments (P > 0.05), and crude fiber (CF) also did not differ significantly (P > 0.05) while exhibiting weak correlations with most physiological indicators. To ensure that PCA focused on the core responsive indicators, these two variables were excluded, and the remaining 20 indicators were retained for PCA, including morphological traits (H, Ln), photosynthetic parameters (Chl, Pn), oxidative stress indicators (O_2_^·^^-^, H_2_O_2_, MDA, REC), antioxidant enzymes (SOD, POD, CAT), osmolyte (Pro), biomass parameters (SFW, RFW, RhFW), and quality traits (DRY, SS, SP, FAA, AsA). The KMO value was 0.827, and Bartlett’s test of sphericity was significant (P < 0.001), indicating acceptable structural validity for PCA. Using the eigenvalue > 1 criterion, one principal component (PC1) was extracted, accounting for 85.172% of the total variance, preliminarily suggesting a consistent coordinated variation trend among these indicators in response to calcium treatment.

#### 3.7.1 Correlation analysis among indicators

Pearson correlation analysis was performed on the 20 indicators (**Figure 7**). The results showed that the 16 positive indicators (Chl, Pn, SOD, POD, CAT, Pro, H, Ln, SFW, RFW, RhFW, DRY, SS, SP, FAA, AsA) were significantly and positively correlated with each other (r ranging from 0.58 to 0.98, P < 0.01), while they were significantly and negatively correlated with the four oxidative stress indicators (O_2_^·^^-^, H_2_O_2_, MDA, REC) (r ranging from −0.57 to −0.98, P < 0.001). The heatmap displayed a distinct orange-green partitioning pattern (orange indicating positive correlations, green indicating negative correlations). These results preliminarily indicated that the 20 indicators exhibited a highly coordinated but opposite response pattern along the calcium concentration gradient, providing a basis for PCA.

**Figure 7:**
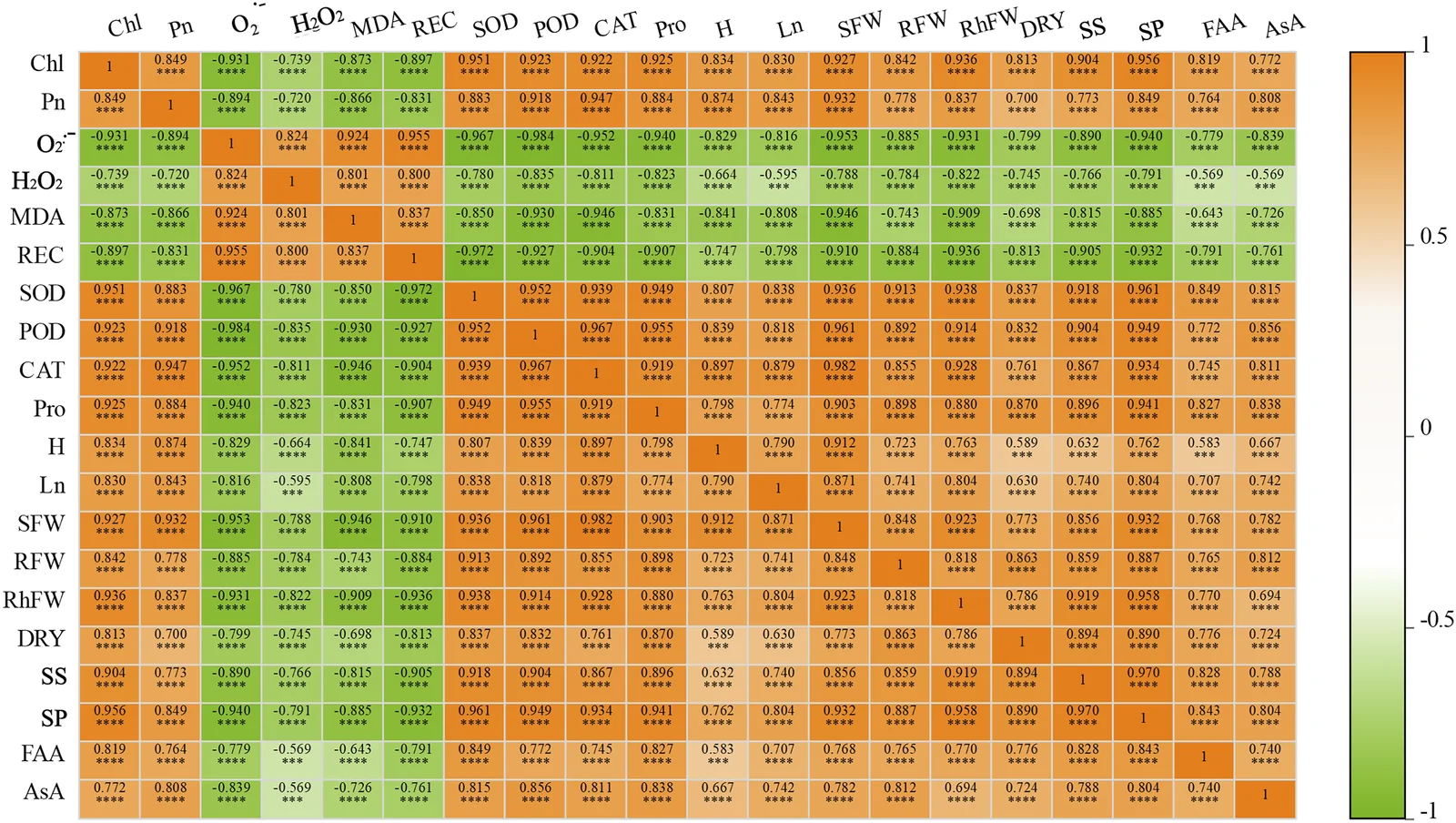
Pearson correlation heatmap of 20 physiological, biochemical, morphological, biomass, and quality indicators in ginger under different calcium nitrate rates. Chl, chlorophyll; Pn, net photosynthetic rate; SOD, superoxide dismutase; POD, peroxidase; CAT, catalase; Pro, proline; H, plant height; Ln, leaf number; SFW, shoot fresh weight; FW, root fresh weight; RhFW, rhizome fresh weight; DRY, dry matter content; SS, soluble sugar; SP, soluble protein; FAA, free amino acid; AsA, ascorbic acid; O_2_^·^^-^, superoxide anion; H_2_O_2,_ hydrogen peroxide; MDA, malondialdehyde; REC, relative electrolyte conductivity. PCA was performed based on the correlation matrix. The Kaiser-Meyer-Olkin (KMO) measure of sampling adequacy was 0.827, and Bartlett’s test of sphericity was significant (P< 0.001). Components with eigenvalues > 1 were extracted. The first principal component (PC1) accounted for 85.172% of the total variance. Asterisks indicate significance levels: *p<0.05, **p<0.01, ***p<0.005, ****p<0.001.

#### 3.7.2 Principal component loadings

As shown in the loading matrix (**Table 1**), PC1 had loadings greater than 0.8 for all 16 positive indicators and less than −0.8 for all four oxidative stress indicators, suggesting strong correlations between each indicator and PC1. Accordingly, PC1 was tentatively designated as the “calcium-regulated integrated effect factor”—with higher scores indicating superior morphogenesis, photosynthetic capacity, antioxidant capacity, biomass, and quality, as well as lower oxidative damage. This naming is proposed based on the current data pattern, and its biological interpretation requires further validation in subsequent studies.

**Table 1:** Principal component loading matrix.

Index	PC1	Index	PC1
Chl	0.958	H	0.844
Pn	0.920	Ln	0.859
O_2_^·–^	-0.826	AgFW	0.972
H_2_O_2_	-0.979	RFW	0.911
MDA	-0.916	RtFW	0.949
REC	-0.951	DRY	0.856
SOD	0.980	SS	0.930
POD	0.984	SP	0.977
CAT	0.975	FAA	0.831
Pro	0.963	AsA	0.843

Chl, chlorophyll; Pn, net photosynthetic rate; H_2_O_2_, hydrogen peroxide; O_2_^·–^, superoxide anion; MDA, malondialdehyde; REC, relative electrical conductivity; SOD, superoxide dismutase; POD, peroxidase; CAT, catalase; Pro, proline; H, plant height; Ln, leaf number; SFW, shoot fresh weight; RFW, root fresh weight; RhFW, rhizome fresh weight; DRY, dry matter content; SS, soluble sugar; SP, soluble protein; FAA, free amino acid; AsA, ascorbic acid. All loadings have absolute values >0.8, indicating strong correlation with PC1.

#### 3.7.3 Comparison of PC1 scores among treatments

One-way ANOVA and Tukey’s HSD multiple comparison test were performed using PC1 score as the integrated indicator. The bar chart (**Figure 8**) showed highly significant differences among treatments (F = 637.361, P< 0.001). PC1 scores followed a trend of initial increase followed by a decline: scores increased significantly from CK through T1, T2, T3, and T4, peaking at T4 (1.399), then decreased significantly in T5 (0.834) compared with T4. Tukey’s grouping preliminarily suggested that T4 was the optimal treatment, followed by T5, with CK being the poorest.

**Figure 8:**
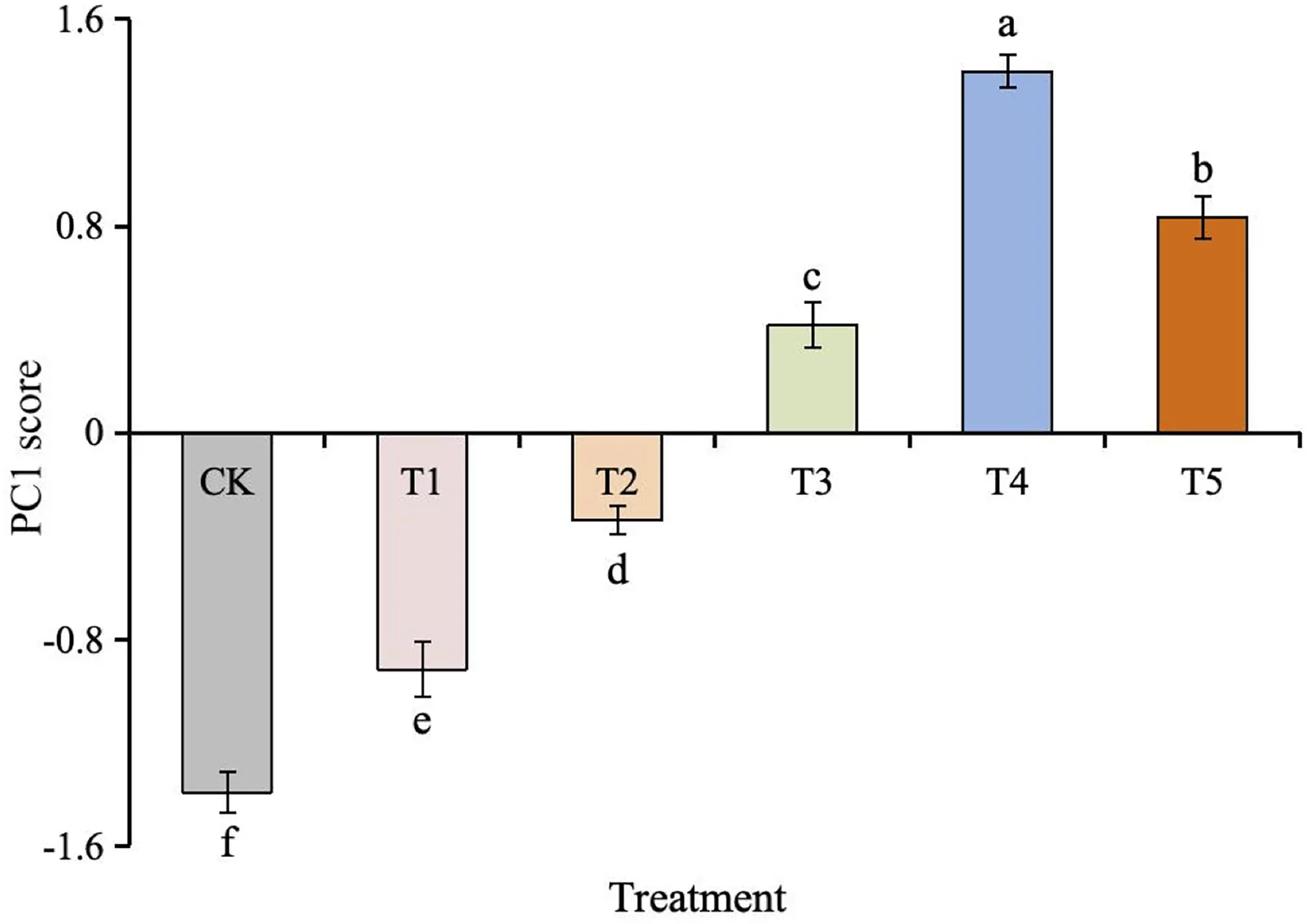
PC1 score bar chart under different Ca(NO_3_)_2_ treatments.Values are means ± SD (n = 4). Different lowercase letters indicate significant differences among treatments (p< 0.05, Tukey’s HSD test). PC1 explained 85.172% of the total variance.

### 3.8 Comprehensive evaluation

We adopted the membership function method to conduct a comprehensive evaluation of morphological, biomass and quality parameters across different Ca(NO_3_)_2_ treatments, with the corresponding results presented in **Table 2**. As shown in **Table 2**, T4 attained the highest average membership degree and came in first place, followed by T5 (second) and T3 (third). The ranking of comprehensive scores across treatments was highly consistent with that of the PC1 scores obtained from PCA (**Figure 7**), which further confirms that T4 is the optimal fertilization dosage.

**Table 2:** Comprehensive evaluation of morphological, biomass and quality of ginger with Ca(NO3)2.

Treatment	H (cm)	S (mm)	L	SW (g)	RF W (g)	RhF W (g)	Dry (%)	SS (mg g^-^¹)	SP (mg g^-^¹)	FAA (mg g^-^¹)	AsA (mg g^-^¹)	CF (%)	D value	Comprehensive ranking
CK	0	0	0	0.08	0	0.08	0	0	0	0	0	0	0.01	6
T1	0.16	0.13	0.32	0.19	0	0.38	0	0.22	0.24	0.11	0	0.25	0.17	5
T2	0.47	0.58	0.58	0.52	0.21	0.51	0.4	0.47	0.43	0.22	0.03	0.5	0.36	4
T3	0.96	0.86	0.8	0.78	0.56	0.74	0.58	0.53	0.67	0.78	0.37	0	0.64	3
T4	1	1	1	1	1	1	1	1	1	1	1	0.75	0.98	1
T5	0.94	0.68	0.9	0.9	0.72	0.84	0.58	0.75	0.76	0.67	0.78	1	0.79	2

H, plant height; S, stem diometer; Ln, leaf number; SFW, shoot fresh weight; RFW, root fresh weight; RhFW, rhizome fresh weight; DRY, dry matter content; SS, soluble sugar; SP, soluble protein; FAA, free amino acid; AsA, ascorbic acid; CF, crude fiber. FW indicates fresh weight. The comprehensive evaluation value (D value) was calculated using membership function analysis; higher D values indicate better overall performance.

## 4 Discussion

### 4.1 Effects of exogenous calcium on photosynthetic characteristics of ginger leaves under natural high-temperature conditions

High temperatures impair protein function and membrane lipid fluidity, thereby compromising chloroplastic enzyme activities and thylakoid membrane stability (Howarth, 2005; Wahid et al., 2007). Under heat stress, chloroplasts suffer structural damage and chlorophyll content declines sharply, leading to reduced photosynthetic efficiency (Salvucci and Crafts-Brandner, 2004; Wahid et al., 2007). Heat stress has also been shown to impair chloroplast protein import and biogenesis, thereby compromising the assembly of photosynthetic complexes (Dutta et al., 2009). In the present study, total chl, chl a, chl b, and Pn in ginger leaves all decreased progressively with extended natural heat exposure, consistent with the findings of Siddiqui et al. (2017) in tomato, further confirming the universal inhibitory effect of high temperature on the photosynthetic system.

Mixed-model analysis revealed an apparent “structure-function decoupling” in the effects of exogenous calcium on the photosynthetic system: the protective effect of calcium on photosynthetic pigments persisted for up to 8–12 d, whereas its promotion of net photosynthetic rate had already substantially diminished by 12 d. This temporal asynchrony suggests that calcium primarily protects photosynthesis through structural maintenance at the light-reaction level (e.g., delaying chlorophyll degradation), but is less effective in preventing heat-induced damage to dark-reaction processes such as carbon assimilation (Yang et al., 2015; Siddiqui et al., 2017). Recent evidence suggests that calcium directly stabilizes photosynthetic machinery by preserving chloroplast ultrastructure and facilitating D1 protein turnover under thermal stress (Faizan et al., 2025). Previous studies have shown that while calcium influx triggered by heat perception at the plasma membrane is an early response event, sustained high temperatures cause membrane lipid peroxidation that disrupts thylakoid membrane structure and inhibits the repair cycle of the PSII reaction center D1 protein, thereby exacerbating photoinhibition (Abdelrahman et al., 2020; Didaran et al., 2024). By 16 d, the regulatory effect of calcium in this study tended to weaken, and the maintenance of the photosynthetic system may have relied more on the plant’s intrinsic basal thermotolerance mechanisms (Abdelrahman et al., 2020; Fang et al., 2025).

### 4.2 Effects of exogenous calcium on reactive oxygen metabolism, membrane damage, and antioxidant defense system in ginger leaves under natural high-temperature conditions

Under high-temperature conditions, the sustained accumulation of reactive oxygen species (ROS) and the consequent cascade of membrane lipid peroxidation ultimately compromise the structural integrity and functional capacity of the plasma membrane system (Samat et al., 2025; Kan et al., 2023). In the present study, both O_2_^·^^-^ release rate and H_2_O_2_ content accumulated progressively with extended natural heat exposure. Mixedmodel analysis revealed that calcium exerted a significantly stronger scavenging effect on O_2_^·^^-^ (P< 0.001) than on H_2_O_2_ (P = 0.046), suggesting that calcium acts more directly and efficiently on upstream ROS. This differential effect may be attributed to the distinct metabolic pathways of these two ROS species in plants (Yang and Poovaiah, 2002; Liang et al., 2011). MDA content and REC, widely recognized as indicators of oxidative membrane damage, both exhibited sustained increases throughout the experimental period (Ma et al., 2018), and the elevation of REC was closely associated with MDA accumulation (Kan et al., 2023). The results obtained in this study are fully consistent with these general patterns, clearly demonstrating that high-temperature conditions imposed significant physiological and biochemical constraints on ginger.

Notably, O_2_^·-^ and H_2_O_2_ contents responded to calcium rate in a linearly decreasing manner (quadratic term P > 0.05), whereas MDA and REC exhibited U-shaped responses with an optimal rate of 60.9 mmol Ca^2+^ plant^-1^. This divergence suggests that although calcium continuously scavenges ROS, the alleviation of membrane damage is subject to a dosage threshold. When the calcium rate exceeded 60.9 mmol plant^-1^, membrane structure and photosynthetic function began to deteriorate despite continued ROS scavenging, possibly due to ionic antagonism or metabolic burden induced by excessive calcium (Liang et al., 2021; Ge et al., 2022).

To cope with oxidative stress induced by high temperatures, plants mobilize their antioxidant enzyme defense network by enhancing the activities of key enzymes such as SOD, POD, and CAT, which synergistically inhibit membrane lipid peroxidation (Samat et al., 2025). Mixed-model analysis revealed that calcium activated SOD, POD, and CAT in a temporally distinct manner rather than synchronously: CAT responded significantly at the early stage (4 d), whereas SOD (12 d) and POD (4–12 d) lagged behind. This temporal sequence reflects the coordinated division of labor among the three enzymes within the ROS scavenging network, with each functioning at different steps and time windows to jointly maintain cellular ROS homeostasis. At the molecular level, the calcium/calmodulin (Ca^2+^/CaM) complex has been shown to directly bind and activate plant catalases, thereby promoting H_2_O_2_ degradation (Yang and Poovaiah, 2002). Previous studies have similarly reported differential enhancements of SOD, POD, and CAT activities by exogenous calcium in other plant species (Fan et al., 2023).

Even though SOD, POD, and CAT activities declined after exceeding the optimal calcium rate, O_2_^·^^-^ and H_2_O_2_ contents continued to decrease linearly. This seemingly contradictory pattern may be explained by the fact that enzyme activity reflects catalytic efficiency per unit time, whereas ROS content represents the cumulative difference between total scavenging and total production over the 16-day measurement period—even if enzyme activity decreased at 76.2 mmol Ca^2+^ plant^-1^, the cumulative scavenging amount may still have exceeded the sustained ROS generation.

Short-term heat stress can induce substantial synthesis of antioxidant enzymes to counteract oxidative damage, whereas prolonged high-temperature exposure exceeds the plant’s tolerance threshold, leading to functional decline and reduced antioxidant enzyme activity—a pattern consistent with observations from this study and others (Sarkar et al., 2016; Gong et al., 2023). In the present experiment, SOD, POD, and CAT activities, as well as proline content, all exhibited inverted U-shaped trends during the early to mid-stage of heat exposure (4–12 d), with the optimal calcium rate converging around 60.9 mmol plant^-1^.

Under high-temperature conditions, plants simultaneously confront multiple stress factors, among which osmotic stress is a concurrent challenge (Samat et al., 2025). The coordinated accumulation of osmotically active solutes effectively reduces cellular water potential, thereby alleviating physiological drought caused by adverse conditions such as high temperature, water deficit, or salt exposure (Ghosh et al., 2021). In this study, proline content increased significantly with calcium application and followed an inverted U-shaped trend, peaking at 12 d, indicating that calcium not only scavenges ROS through enzymatic antioxidant systems but also maintains cellular turgor and water balance by promoting the accumulation of osmotic regulators such as proline. This synergistic “enzymatic ROS scavenging + osmotic adjustment” mechanism constitutes a dual protective strategy by which exogenous calcium alleviates heat-induced damage (Feng et al., 2023), a temporal pattern also reported in other species (Raza et al., 2023).

The protective role of calcium in safeguarding photosynthetic organs—by reducing oxidative load and enhancing membrane stability—has been well documented (Navazio et al., 2020; Wang et al., 2022; Fan et al., 2023; Feng et al., 2023). The realization of these protective effects is associated with the activation of antioxidant enzyme systems and effective inhibition of membrane lipid peroxidation. In the present study, calcium application significantly reduced O_2_^·^^-^ accumulation and H_2_O_2_ content, upregulated SOD, CAT, and POD activities, and concurrently lowered MDA and REC levels, suggesting that calcium may contribute to heat tolerance in association with activation of the antioxidant defense system and mitigation of membrane damage. Among all treatments, the 60.9 mmol Ca^2+^ plant^-1^ rate consistently performed best across all indicators. Correspondingly, the effectiveness of Ca(NO_3_)_2_ in enhancing terminal heat tolerance through strengthened antioxidant defense and osmolyte accumulation has been validated in multiple plant species (Siddiqui et al., 2017; Wang et al., 2023; Guo et al., 2023).

### 4.3 Optimal calcium rate and effective time window for alleviating heat-induced damage in ginger

Synthesizing all physiological indicators measured during the seedling stage, the alleviating effect of exogenous calcium on ginger under natural high-temperature conditions exhibited highly consistent dose-and time-dependent patterns. In terms of dosage, photosynthetic rate and chlorophyll content followed an inverted U-shape, MDA and REC followed a U-shape, and SOD, POD, CAT, and proline followed an inverted U-shape, with the optimal calcium rate for all four categories converging around 60.9 mmol plant^-1^. In terms of temporal dynamics, the threshold effect for all indicators was most pronounced during the early to mid-stage of heat exposure (4–12 d) and gradually diminished by 16 d.

Previous reviews have noted that the activation of plant heat-tolerance defense systems follows a clear temporal hierarchy—early response mechanisms are rapidly activated at the onset of stress, but their efficiency gradually decays as stress persists (Qu and Yamori, 2026)—a pattern closely matching the “effective at 4–12 d, weakened by 16 d” temporal window observed in this study. In the present study, the 4–12 d period coincided with the peak accumulation of antioxidant enzyme activities (SOD, POD, CAT) and osmotic regulators (proline). Calcium effectively suppressed membrane lipid peroxidation (as indicated by reduced MDA and REC) and maintained photosynthetic function calcium application was associated with suppressed membrane lipid peroxidation … and maintained photosynthetic function, coinciding with the activation of these defense systems. By 16 d, however, irreversible damage accumulated from prolonged heat exposure intensified, antioxidant enzyme activities declined, and ROS scavenging capacity deteriorated, collectively contributing to the attenuation of calcium’s protective effects—indicating that calcium regulation operates within a defined temporal window of effectiveness.

Our observed temporal window of calcium efficacy (4–12 d) is consistent with the conceptual framework proposed by Qu and Yamori (2026), who emphasized that plant heat-tolerance defense systems operate under a strict temporal hierarchy, with early response mechanisms activated rapidly but decaying progressively as stress persists. Within this framework, our findings demonstrate that the 4–12 d window coincides with the peak activity of enzymatic antioxidants (SOD, POD, CAT) and osmotic regulators (proline), during which calcium application effectively suppressed membrane lipid peroxidation and maintained photosynthetic function. By 16 d, however, the cumulative damage from prolonged heat exposure exceeded the capacity of these defense systems, leading to the attenuation of calcium’s protective effects. This temporal alignment across independent studies suggests that the 4–12 d window may represent a conserved phase of calcium-mediated thermoprotection in thermophilic horticultural crops, providing a practical reference for optimizing the timing of exogenous calcium application in heat stress management.

### 4.4 Effects of different Ca(NO_3_)_2_ rates on morphological and biomass parameters of ginger

In terms of morphogenesis, exogenous calcium has been shown to significantly promote morphological traits such as plant height and leaf number in tomato (Haider et al., 2024) and adzuki bean (Lian et al., 2022). Consistently, the present study found that Ca(NO_3_)_2_ application significantly promoted plant height and leaf number in ginger, whereas no significant promoting effect was observed on stem diameter.

Previous studies have demonstrated that exogenous calcium supplementation positively promotes yield and biomass accumulation in various crops (Coelho et al., 2022; Kang et al., 2024; Zhang et al., 2024). Similar enhancement of belowground sink organs by calcium has been reported in potato, where supplemental calcium fertilization increased tuber size and number (Ozgen et al., 2003). At the photosynthetic and physiological level, calcium can regulate photosynthetic carbon assimilation efficiency and promote the translocation of photosynthetic products to sink organs (Feng et al., 2023; Wang et al., 2023), which may represent one of the key physiological bases for calcium-induced yield and biomass enhancement. Source-sink relationships are fundamentally modulated by mineral nutrient availability, with calcium playing a critical role in phloem loading and assimilate partitioning (Kirkby et al., 2023). In this study, SFW, RFW and RhFW all reached their maximum values under T4, representing increases of 116.2%, 56.0%, and 62.7% over the control, respectively, indicating that calcium significantly promoted biomass accumulation in both aboveground and belowground parts of ginger. Among these, SFW exhibited the greatest magnitude of increase, suggesting that the promoting effect of calcium was first manifested as enhanced aboveground photosynthetic capacity (expansion of the “source”), which in turn drove rhizome enlargement (filling of the “sink”) (Venkateswarlu and Visperas, 1987; Deng et al., 2025). SFW was highly significantly and positively correlated with rhizome fresh weight (r = 0.923, P< 0.01). At T5, all parameters declined slightly compared with T4 (though remaining significantly higher than the control), indicating that the promoting effect of calcium tended to weaken beyond the optimal concentration, reflecting a dose-saturation effect (Marschner, 2012). This is consistent with findings in rose (Wang et al., 2022) and Rosa (Salemi et al., 2025), suggesting that the promoting effect of exogenous calcium on plant growth exhibits a conserved concentration threshold—moderate calcium promotes growth and development, whereas excessive calcium diminishes this effect. The attenuation at supra-optimal concentrations may be attributed to osmotic stress induced by high calcium levels or antagonistic interactions with K^+^ and Mg^2+^ (Wu et al., 2025).

### 4.5 Optimal calcium rate and effective time window for alleviating heat-induced damage in ginger

In the present study, all quality parameters were significantly improved under T4 compared with CK. Among these, soluble protein was the most responsive to calcium application (+36.5%, P< 0.001), which may be attributed to the role of calcium as a signaling molecule in activating protein kinases and regulating the expression of protein synthesis-related genes (Thor, 2019). Soluble sugar (+28.1%), free amino acids (+19.1%), and vitamin C (+17.5%, reaching 2.15 mg/g) were also significantly enhanced, with their accumulation closely associated with photosynthetic product translocation (Kleinhenz and Palta, 2002), nitrogen metabolism regulation, and ascorbate biosynthesis, respectively (Tuna et al., 2007; Smirnoff and Wheeler, 2000). Consistent with our findings, similar calcium-induced enhancements of soluble sugar, protein, and vitamin C contents have been reported in tomato (Haleema et al., 2024) and mini Chinese cabbage (Yang et al., 2025) under stress conditions. Dry matter content reached 10.58%, representing a 3.6% increase over the control, indicating that the promoting effect of calcium fertilization resulted from effective dry matter accumulation rather than increased water uptake—a finding of positive significance for improving commercial value and processing suitability.

Notably, all parameters at T5 showed slight declines compared with T4, though they remained significantly higher than the control, indicating that the promoting effect of calcium tended to weaken beyond the optimal concentration, reflecting a dose-saturation effect. This dose -saturation pattern is consistent with the observations of Yang et al. (2025) in mini Chinese cabbage, where calcium levels beyond the optimum resulted in diminished nutritional quality—in line with the dose-saturation pattern described above.

Furthermore, crude fiber content did not differ significantly among treatments (P = 0.926), indicating that Ca(NO_3_)_2_ application did not cause rhizome lignification or textural deterioration, thereby supporting the feasibility of calcium Ca(NO_3_)_2_ application in ginger production from a palatability perspective.

In summary, calcium application improved ginger quality through a comprehensive synergistic enhancement of dry matter content, SS, SP, FAA, and AsA, although this promoting effect operated within an optimal concentration range, beyond which the enhancement diminished. Crude fiber content remained unaffected, suggesting that calcium fertilization can effectively improve the nutritional quality of rhizomes without compromising palatability.

### 4.6 Comprehensive evaluation based on correlation analysis and principal component analy

The Pearson correlation heatmap (**Figure 6**) revealed that the 16 positive indicators—Chl, Pn, SOD, POD, CAT, Pro, H, Ln, SFW, RFW, RhFW, dry matter content, SS, SP, FAA, and AsA—were significantly and positively correlated with each other (r ranging from 0.59 to 0.98, P< 0.01), while they were significantly and negatively correlated with the four oxidative stress indicators (O_2_^·^^-^, H_2_O_2_, MDA, REC) (r ranging from −0.57 to −0.98, P< 0.001). The heatmap displayed a distinct orange-green partitioning pattern. This highly differentiated correlation pattern suggests that, under calcium treatment, morphogenesis, photosynthetic capacity, antioxidant enzyme activities, osmotic adjustment, biomass accumulation, and quality traits in ginger were associated synergistic promoting relationships, whereas the degree of oxidative damage varied inversely with these positive indicators—a finding consistent with previous studies under heat stress (Hasanuzzaman et al., 2014; Siddiqui et al., 2017). The correlation analysis provided a structural basis for PCA.

The PCA loading matrix (**Table 1**) further illustrated the contribution weights of each indicator to the integrated effect. PC1 had loadings greater than 0.8 for all 16 positive indicators and less than −0.8 for all four oxidative stress indicators, which was highly consistent with the correlation analysis results. The ranking of PC1 scores (**Figure 1b**) and the distribution of treatments along the PC1 axis (CK< T1< T2< T3< T5< T4) revealed a non-linear characteristic of the calcium fertilization effect: low calcium rates (T1, T2) did not result in significant improvement, moderate-to-high calcium rates (T3, T4) significantly enhanced overall performance, with T4 peaking, whereas T5 (excessive calcium) declined slightly compared with T4 but still outperformed the low-calcium treatments. This “low-optimal-slight decline” parabolic effect is consistent with findings in rose (Wang et al., 2022) and tomato (Ayub et al., 2022) under heat stress, indicating that appropriate exogenous calcium supply can alleviate heat stress by enhancing membrane stability, maintaining photosynthetic efficiency, and sustaining antioxidant capacity.

The membership function analysis (**Table 2**) further quantified the promoting effects of each treatment on actual growth and quality. The ranking of comprehensive scores (T4 > T5 > T3 > T2 > T1 > CK) was fully consistent with the PC1 score ranking. This analysis included only growth morphology, biomass, and quality indicators directly related to yield and quality, independently validating the conclusion that T4 was optimal in the PCA model. The three analytical approaches—correlation analysis, PCA loading matrix, and membership function comprehensive scoring—corroborated each other, consistently confirming that T4 was the optimal calcium fertilizer rate under the experimental conditions. Appropriate calcium supply synergistically enhanced ginger growth, yield, and quality performance under heat stress by strengthening antioxidant capacity, maintaining membrane stability, and promoting photosynthetic carbon assimilation.

### 4.7 Limitations

Due to the limited sample size (24 samples), PCA was employed as an exploratory data analysis tool rather than as the sole basis for confirmatory statistical inference. The variable-to-sample ratio (approximately 1:1.2) falls below the recommended threshold for multivariate analysis, and the condensation of 20 indicators into a single principal component, while effectively reducing dimensionality, inevitably sacrifices detailed information on individual indicator variation. Therefore, the PCA results are intended to serve as a reference for comprehensive evaluation rather than as stand-alone evidence.

Several additional limitations should be acknowledged. The HSI was calculated based on daily maximum temperatures (Ti > 35 °C) without accounting for the duration of high-temperature exposure, and the AUC integration employed equal calendar intervals, which may not fully reflect the actual heat load experienced by plants under natural conditions. Future studies with multi-year trials, expanded sample sizes, and refined temperature integration methods are warranted to further validate the threshold and temporal dynamics of calcium efficacy. While our results demonstrate significant associations between calcium application and improved physiological performance under high-temperature stress, this study primarily relied on phenotypic and biochemical indicators. We did not directly measure upstream signaling events such as calcium flux, calmodulin activation, or downstream molecular responses including heat shock protein expression or stress-responsive gene transcription. Therefore, the mechanistic interpretations provided in this discussion—while consistent with the observed correlations—should be regarded as suggestive rather than definitive. Future studies incorporating calcium-specific fluorescent probes, pharmacological inhibitors, or transcriptomic analyses are needed to establish the precise molecular pathways through which exogenous calcium enhances thermotolerance in ginger.

Moreover, this study was conducted in a single growing season (2023) at one location (Shouguang, Shandong) using one ginger cultivar (‘Laiwu Big Ginger’). The optimal rate identified (60.9 mmol Ca^2+^ plant-1) should be interpreted within these specific conditions and may not be directly generalizable to other environments, soil types, or production systems. Multi-site and multi-year field validations are needed before any broader agronomic recommendations can be made.

## 5 Conclusion

Exogenous Ca(NO_3_)_2_ effectively alleviates heat injury in ginger under natural high-temperature conditions and this effect is associated with enhanced photosynthetic capacity, activated antioxidant enzymes, reduced ROS accumulation, and maintained membrane stability These physiological adjustments promoted vegetative growth and rhizome nutritional quality, with optimal performance at 60.9 mmol Ca^2+^ plant^-1^. PCA and correlation analyses revealed a synergistic network associated with calcium-mediated thermotolerance. These findings suggest that exogenous calcium supplementation may serve as a practical strategy to sustain ginger production under naturally elevated temperatures.

## Data Availability

The raw data supporting the conclusions of this article will be made available by the authors, without undue reservation.
